# Video Laryngoscopes in Simulated Neonatal Intubation: Usability Study

**DOI:** 10.3390/children12060723

**Published:** 2025-05-31

**Authors:** Jasmine Antoine, Kirsty McLeod, Luke Jardine, Helen G. Liley, Mia McLanders

**Affiliations:** 1 Mater Mothers’ Hospital, Mater Research, The University of Queensland, St Lucia 4072, Australia; hliley@uq.edu.au; 2 Clinical Skills Development Service, Metro North Health, The University of Queensland, St Lucia 4072, Australia; kirsty.mcleod@health.qld.gov.au (K.M.); mia.mclanders@health.qld.gov.au (M.M.); 3 Mater Mothers’ Hospital, The University of Queensland, St Lucia 4072, Australia; luke.jardine@mater.org.au

**Keywords:** tracheal intubation, human factors, human–computer interaction, usability testing, newborn, infant

## Abstract

**Background/Objectives**: Neonatal intubation is a complex procedure, often associated with low first-pass success rates and a high incidence of complications. Video laryngoscopes provide several advantages, including higher success rates, especially for novice clinicians, a magnified airway view that can be shared with supervisors, and the ability to record still or video images for debriefing and education. However, video laryngoscope devices vary, raising the possibility of differences in usability. **Methods**: The study used mixed methodology, including observations, semi-structured interviews, think-aloud techniques, high-fidelity simulations, function tests, and questionnaires to assess usability, defined by the clinician satisfaction, efficacy, and efficiency of six video laryngoscope devices; (1) C-MAC^®^ with Miller blade, (2) GlideScope^®^ Core^TM^ with Miller blade, (3) GlideScope^®^ Core^TM^ with hyperangle LoPro blade, (4) Koala^®^ Vision Ultra with Miller blade, (5) Koala^®^ Handheld with Miller blade, and (6) Parker Neonatal with Miller blade. Clinician satisfaction was determined by the System Usability Scale (SUS), National Aeronautics and Space Administration Task Load Index (NASA-TLX), and clinician preference. Device efficacy was determined by first-pass success, number of attempts, and overall success. Efficiency was assessed by time to successful intubation and function test completion rates. **Results**: Neonatal video laryngoscopes varied considerably in design, impacting usability. All devices were deemed suitable for neonatal intubation, with the Koala^®^ Handheld, C-MAC^®^, and GlideScope^®^ Core ^TM^ Miller demonstrating the highest usability. **Conclusions**: This simulation-based study highlights substantial variability in neonatal video laryngoscope usability, indicating the need for further research into usability in the clinical setting.

## 1. Introduction

Neonatal intubation is a difficult, complex procedure with low rates of first-pass success and high rates of complications [1]. Traditionally, neonatal intubation was performed using a conventional laryngoscope, a handheld device with blade and built-in light source that allow visualization of the vocal cords via direct line of sight to guide the placement of a tracheal tube. For the purposes of this study, we will refer to these devices collectively as conventional laryngoscopes, although the terms ‘traditional laryngoscope’ and ‘direct laryngoscope’ are also used in the literature. Video laryngoscopes are devices that combine a built-in light source and a small camera on the blade to project a magnified view of the vocal cords onto a screen. Many can also be used to provide a direct line-of-sight view of the vocal cords, giving the intubating clinician the flexibility to choose between direct or video-assisted views during intubation. The use of video laryngoscopes in neonatal intubation is increasing [2]. The benefits of video laryngoscopy include magnification of the airway view, reduced need to achieve direct line of sight of the vocal cords, and the ability for other team members, including supervisors, to concurrently see what the person performing the intubation is seeing. Systematic reviews have indicated that video laryngoscopy compared to conventional laryngoscopy improves first-pass success and may reduce adverse events [3,4,5]. The International Liaison Committee on Resuscitation (ILCOR) has recently suggested the use of video laryngoscopy in neonatal intubation, particularly for less-experienced clinicians [6]. Most devices also allow recording of still or video images for later debriefing and education [7,8]. Video recordings are a useful form of objective evidence, as memories of events can vary, especially under stressful conditions, and may be subject to individual recall biases [9]. The review of video recordings of neonatal resuscitation for debriefing is well established and may improve outcomes [10]. More specifically, the use of images from video laryngoscopes can provide insights into unsuccessful attempts [11].

The device characteristics of neonatal video laryngoscopes vary [7,12,13]. Differences include the size of the video screen and whether the screen is fixed to the blade and handle or detached. Detached screens can be mounted on a stand or remain unmounted. Video laryngoscope blades vary in size, shape, integration with the handle, and reusability (single or reusable). Magnification and the method of recording images also vary. These characteristics affect the way that clinicians interact with the device, referred to as user interface, and the appropriateness for different infant populations. The design of a video laryngoscope device can impact the clinician’s subjective experience, including the perceived level of physical demand, cognitive workload, and ease of learning [14]. Usability refers to the device characteristics and user interface elements that facilitate clinicians’ achievement of the key functions of video laryngoscopes, including intubation, recording of intubations, and troubleshooting device failure. This study explores video laryngoscope usability by assessing user satisfaction, efficacy, and efficiency [15,16]. Despite evidence that in neonatal intubation, video laryngoscope device characteristics affect clinician experience, we found no previous studies that had specifically examined the usability of different devices.

Human-centered design is important in ensuring that clinical devices optimally meet all performance criteria, and simulation testing can be well suited to evaluating the interaction of users with multiple devices in a controlled setting without risk to patient safety [17]. Given the potential value of video laryngoscopes in neonatal intubation, we saw value in investigating the various devices to determine which of them most effectively support clinicians.

We hypothesized that six video laryngoscope devices from four different manufacturers, when used in simulated neonatal intubation, would have variations in usability, measured as clinician satisfaction, efficacy, and efficiency.

## 2. Materials and Methods

### 2.1. Study Design

**Figure 1:**
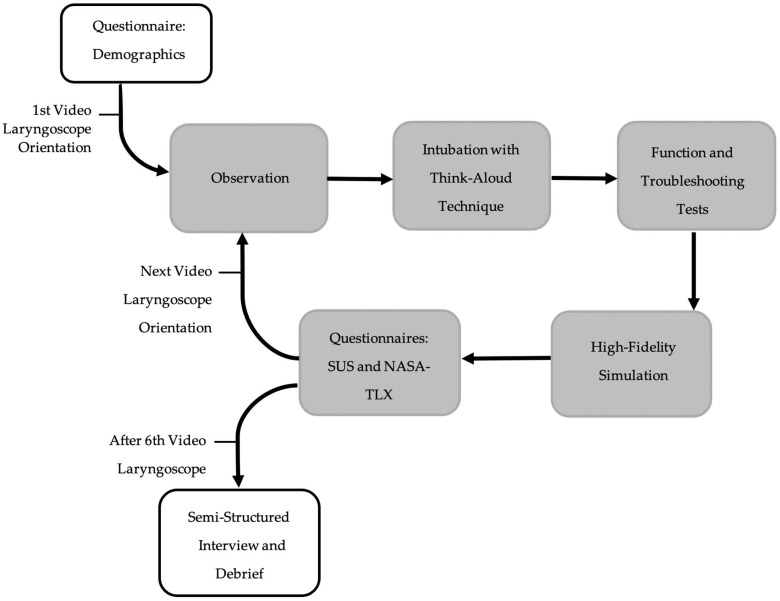
Study procedure flowchart.

We evaluated the usability of six video laryngoscope devices currently marketed in Australia, in simulated neonatal intubation using mixed methodology. Participants first completed a questionnaire that addressed demographics and their prior intubation experience with conventional and video laryngoscopes. The order of device use was randomly assigned using a computer-generated sequence, with allocation concealed until the first video laryngoscope. The study procedure is outlined in Figure 1. For each device, participants received device orientation. They then were observed using each device. Participants performed an intubation using the think-aloud technique, with their comments recorded for later thematic analysis. Next, participants completed a series of function and troubleshooting tests to evaluate the user interface, functionality and potential device failure. In the next phase, participants undertook a high-fidelity simulation of an apnoeic term baby in a birth suite setting, with an assistant. This simulation was used to assess first-pass success, number of attempts, overall success, and time to successful intubation. If a participant was unable to successfully intubate with a particular device during the initial phase, they were not asked to complete the high-fidelity simulation using that device. In such cases, outcome measures including first-pass success, number of attempts, and overall success were derived from the corresponding think-aloud intubation attempts. Upon completion of testing, participants completed two questionnaires, the System Usability Scale (SUS) [18] and National Aeronautics and Space Administration Task Load Index (NASA-TLX) [19]. Following the experiments, participants completed a semi-structured interview regarding preferred devices. The complete details of the study procedure are provided in Supplementary Material S1.

The study was conducted according to the guidelines of the Declaration of Helsinki and approved by the Metro North Health Human Research Ethics Committee (HREC/2022/MNHA/90362, date of approval, 16th November 2022). Informed consent was obtained from all participants involved in the study.

### 2.2. Setting and Participants

Participants were a convenience sample of consultant neonatologists. The target sample size was five participants, based on established usability research indicating that this number detects the majority (80%) of usability issues, and including more participants is unlikely to provide new insights [20,21]. Consultant (attending) neonatologists were targeted as participants because of their proficiency in neonatal intubation [22], allowing them to focus on evaluating the device, rather than focusing on the procedure. Additionally, since consultant neonatologists have responsibilities for supervision and training, their experiences are valuable to explore.

The study was conducted in a simulation laboratory that replicated the clinical setting of a neonatal resuscitation station in a birthing suite. This setting was simulated because it is one of the common sites of neonatal intubation [23].

### 2.3. Equipment

Six video laryngoscope devices from four different manufacturers were included in testing: (1) C-MAC^®^ with Miller blade (C-MAC) (KARL STORZ, Brisbane, Australia), (2) GlideScope^®^ Core^TM^ with Miller blade (GlideScope Miller) (Verathon, Brisbane, Australia), (3) GlideScope^®^ Core^TM^ with hyperangle LoPro blade (GlideScope LoPro) (Verathon, Brisbane, Australia), (4) Koala^®^ Vision Ultra with Miller blade (Koala Vision) (Koala Medical, Sydney, Australia), (5) Koala^®^ Handheld with Miller blade (Koala Handheld) (Koala Medical, Sydney, Australia), and (6) Parker Neonatal (Parker) (Parker Healthcare, Melbourne, Australia). Intubations were performed on term infant SimNewB^®^ (Laerdal Medical, Brisbane, Australia) manikin. A term manikin was used because, although extremely preterm, very preterm, and late preterm infants are at higher risk of needing intubation, term infants comprise the largest numbers of intubated infants [23].

### 2.4. Outcome Measures

The usability of video laryngoscope devices was determined by clinician satisfaction, efficacy, and efficiency.

Clinician satisfaction was determined by analysis of clinicians’ preferences, the SUS, and NASA-TLX. The SUS is a validated tool to measure the ease of use, learnability, and clinician’s confidence in a device, with scores out of 100 and higher scores indicating better usability [18]. The NASA-TLX is a measure of perceived workload via mental, physical, and temporal demand, performance, effort, and frustration, with each domain scored out of 100 [19]. Lower NASA-TLX scores indicate lower perceived workload [19].

Efficacy of the video laryngoscope devices was assessed by first-pass success, total number of attempts, and overall success in the high-fidelity simulation. First-pass success was defined as intubation following the initial insertion of the laryngoscope blade into the mouth. Each introduction of the laryngoscope blade into the mouth was counted as an intubation attempt. Overall success was defined as successful intubation within four attempts.

Efficiency of the video laryngoscope devices was assessed by the time to successful intubation in the high-fidelity simulation and rates of completion for the function and troubleshooting tests. The time to successful intubation was defined as the time taken from cessation of intermittent positive pressure ventilation (IPPV) via the mask until commencement of IPPV via the tracheal tube (TT) for the successful intubation [24]. Function and troubleshooting tests included participants’ ability to change the laryngoscope blade, intubate under a direct view of the airway, ability to take a photo and record video footage, whether participants would check the video output screen prior to inserting the blade into the mouth, and a device failure test. Device failure may include failure of projection to the video output screen or the camera view becoming obscured by secretions or misting. In this study, we discuss device failure as the video output screen going black while the light source continued to work.

### 2.5. Statistics and Analysis

Data were analysed using IBM SPSS statistics version 29.0 (IBM Corp., Armonk, NY, USA). The measured data for nominal and ordinal variables are expressed as a frequency and percentage. For continuous variables, data are expressed as median and interquartile range (IQR). The experiments were audio-recorded, and participant comments were transcribed for thematic analysis.

## 3. Results

### 3.1. Participant Demographics

Participants were five consultant neonatologists with varied experience using video laryngoscopes in neonatal intubation (Table 1). The majority (60%) of participants had performed fewer than five intubations with video laryngoscopes. Two participants reported prior experience with four of the devices; however, no participants had prior experience with all the tested devices. Two participants had no experience with any of the devices.

### 3.2. Device Characteristics

Each of the six video laryngoscope devices was evaluated for blade size, shape, and reusability, as well as screen size and mounting characteristics (Table 2). Of the six devices, two had screens that were unmounted, three had screens mounted on a stand with the GlideScope devices having an articulated arm, and the Koala Handheld device had the screen mounted to the handle and blade. All devices except the GlideScope LoPro had Miller blades. Only the Koala devices and Parker had Miller 00 blades. The key themes of participant comments focused on the ergonomics of the handle and blade, screen visibility and mounting, teaching utility, and comparison to conventional laryngoscopes (Table 2).

### 3.3. Clinician Satisfaction

The SUS scores varied, with the Parker scoring lowest (median: 32.5) and the Koala Handheld scoring highest (median: 87.5) (Table 3). A SUS score of above 68 is interpreted as indicating above average usability [25]; however, in this study, only three devices met this criterion.

The NASA-TLX scores for perceived workload differed widely between video laryngoscope devices (Table 4). The device with the lowest perceived workload was the C-MAC (median: 26.7), and the Parker had the highest overall perceived workload (median: 50.8).

In a semi-structured interview, when asked to nominate their overall preferred video laryngoscope device, most participants (60%) nominated the Koala Handheld video laryngoscope. These preferences were based on ease of use, ergonomic handling, availability of 00 Miller blade suitable for neonates less than 500 g, and its design similarity to conventional laryngoscopes. For teaching purposes, most participants (60%) said they would prefer the Koala Handheld device. Other advantages of the Koala Handheld device noted were the ease of obtaining a direct view, similarity to a conventional laryngoscope with Miller blades, as well as the location of the video output screen, which was within the same field of vision as that needed to see the baby while intubating. The two participants who said they would prefer other devices for teaching noted the small video screen of the Koala Handheld and potential challenges of ensuring both trainee and supervisor could see the screen. In the semi-structured interview, when asked to consider cases of a known difficult airway, 60% of participants stated that they would prefer a conventional laryngoscope for intubation. Although there was no consensus for any one video laryngoscope device for a difficult airway, 40% selected the GlideScope LoPro. Participants reported on the ease of obtaining a view with the GlideScope LoPro, stating it “followed natural curve of airway, best functional blade”(P4), which may be beneficial for newborns with difficult airways.

### 3.4. Efficacy

All participants had first-pass success with three video laryngoscopes devices: Parker, C-MAC, and Koala Handheld (Table 5). The median number of intubation attempts was the same for all video laryngoscope devices. However, one participant forgot to turn on two devices (the Koala Vision and GlideScope Miller) prior to inserting the blade into the mouth and hence required two attempts to intubate. A further participant forgot to turn on the Koala Handheld device but realised prior to inserting the blade into the mouth, so this was not counted as an attempt. During the initial think-aloud technique intubation, one participant was unable to intubate with the GlideScope LoPro after four attempts and therefore was not asked to proceed to the high-fidelity simulation with this device.

### 3.5. Efficiency

The times to successful intubation are reported in Table 5, with the results for the GlideScope LoPro omitting one participant who was not able to intubate with this device. The median time to successful intubation varied, with participants intubating fastest with the C-MAC (median 23.0s) and slowest with the GlideScope LoPro (median 35.5s). Participants highlighted that the GlideScope LoPro required a different intubation technique due to the shape of the blade: “Intubation with this is very different technique… Have to shape ETT in shape of hockey stick… Not conventional intubation… See beautiful view but can’t pass the ETT”. (P3)

**Figure 2:**
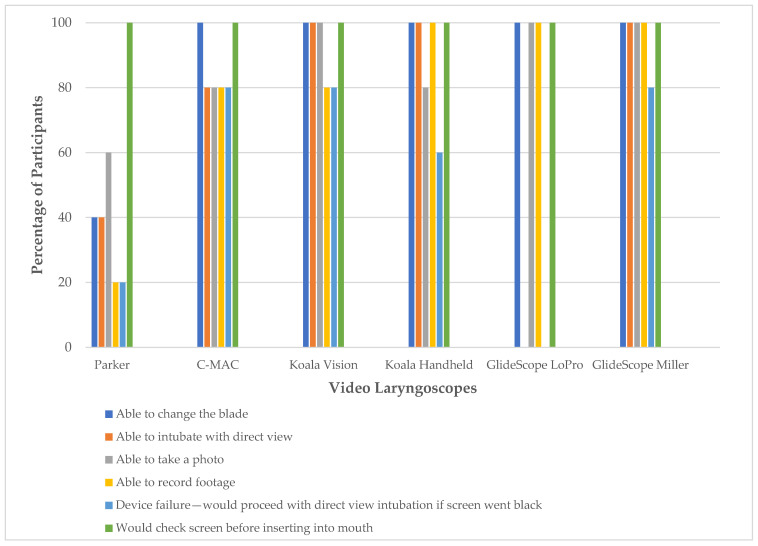
Percentage of participants able to complete function and troubleshooting tests.

The results of the function and troubleshooting tests are shown in Figure 2. The Parker device received the lowest functionality ratings, with most participants unable to change the blade, record footage, or obtain a direct view. Blade replacement was commonly identified as non-intuitive, highlighting the need for explicit instruction. No participants were able to obtain a direct airway view with the GlideScope LoPro, noting the hyperangle of the blade. Although all participants indicated they would verify the video screen before blade insertion, this step was inconsistently performed during the high-fidelity simulations.

## 4. Discussion

This study evaluated six neonatal video laryngoscope devices and and found that variations in device design affected their usability during simulated neonatal intubation. Overall, the Koala Handheld, C-MAC, and GlideScope Miller demonstrated the highest usability, followed by the Koala Vision, GlideScope LoPro, and Parker devices. The Koala Handheld device had an efficient time to intubation, the highest usability score (SUS), and a low perceived workload (NASA-TLX) score. The C-MAC had the fastest time to intubation and the lowest perceived workload score, but this device had lower-than-average SUS scores. The GlideScope Miller had a faster-than-average time to intubation and a high usability score; however, one participant required two attempts to successfully intubate. All participants were able to intubate on the first pass with the C-MAC, Koala Handheld, and the Parker device. In our study, most participants preferred the Koala Handheld for their personal use and considered it the preferred device when supervising novice clinicians, citing its usability as key factors in their preferences. As video laryngoscopes become an increasing integrated into neonatal care, understanding how device design features influence usability is critical to training effectiveness, clinician experience, and clinical outcomes.

The GlideScope LoPro presented notable challenges for participants in this study during simulated neonatal intubation. The hyper-angulated blade design of the GlideScope LoPro was reported to alter intubation technique, with the potential to increase the difficulty of the procedure. Additionally, the design of the GlideScope LoPro device does not allow intubation under direct vision. The inability to perform intubation under direct vision limits the user’s options and adaptability during the procedure. One participant was unable to successfully intubate using this device. For those who were able to intubate, the GlideScope LoPro device had the slowest time to successful intubation. These findings suggest that the GlideScope LoPro may present usability challenges when considering a broader range of clinicians, especially novices who may have difficulties with the hyper-angulated blade design. While participants noted the ease of obtaining a view with this device, the hyper-angulated blade presented some difficulties in inserting the tracheal tube. This blade may of be of utility for infants with congenital anomalies, whose airways may not be able to be visualised with a straight Miller blade. This variability in usability highlights the importance of considering clinician experience, device familiarity, and the intended patient population when selecting devices.

This study demonstrates that variations in design of neonatal video laryngoscope devices affect usability. In a short review on neonatal video laryngoscopes, Kirolos and O’Shea [13] suggested that uniformity of video laryngoscopes and similarity to conventional laryngoscopes might improve the consistency of clinician performance. Although design similarity may offer familiarity to clinicians, device design should prioritise usability by clinicians of varying experience and for a diverse patient population. Neonates who require tracheal intubation range from extremely preterm to post-term and include infants with variations and anomalies of airway anatomy, conferring difficulties that video laryngoscopy may partly resolve. Familiarity of the user experience may enhance performance when clinicians need to use an alternative device (e.g., if they work in more than one location), and in these situations, the resemblance to conventional laryngoscopes may facilitate switching between video and conventional devices. However, where there are similarities between conventional and video laryngoscopes in device ergonomics, the cognitive and perceptual requirements for intubation will differ if the intubator is using the video screen to guide tracheal placement. We suggest that ultimately, video laryngoscope device design that enables high usability may be more important than uniformity in design.

As video laryngoscopy becomes more common in neonatal intubation, the need for device specific training must be considered. In this study, the Parker device demonstrated lower usability in simulated neonatal intubation. Only 40% of participants were able to perform the critical task of changing a blade on the Parker device, with multiply participants unable to complete most of the function tests. Devices with more intuitive designs facilitated better performance in troubleshooting and function tests. These results highlight the importance of intuitive design and the necessity for additional training before clinical use of less-intuitive devices.

This study also identified a need for training in managing device failure. The potential for device failure is not unique to video laryngoscopes, as conventional laryngoscopes can have failures of battery or bulb, preventing illumination of the airway. With the five video laryngoscope devices capable of providing a direct airway view, participants were often reluctant to proceed with intubation when the video screen failed, even if a direct view was available. For four devices, a higher proportion of participants were able to obtain a direct view than felt confident to proceed when the screen failed. This finding highlights that importance of equipping clinicians with the knowledge of a device, its functionality and the ability to troubleshoot problems including screen or device failure. These results align with the recent ILCOR recommendations suggesting the use of video laryngoscopes, particularly for less-experienced clinicians, but that a conventional laryngoscope device needs to be available as a back-up device [6].

Extremely preterm infants are more likely to require intubation than those of higher gestation and more often require multiple attempts to successfully intubate [26]. Intubating extremely premature infants is difficult for several reasons. From a usability perspective, a key aspect is the availability of appropriately sized blades. All devices reviewed in this study had size 0 blades. However, only three devices, the Parker, Koala Vision, and Koala Handheld, had 00 Miller blades suitable for an infant of fewer than 26 weeks gestational age. The Parker device also had a Miller 000 blade. The availability of appropriately size blades for the smallest infants may need to be addressed for those video laryngoscopes that have a more restricted range of blade sizes. Further research exploring the usability of the video laryngoscope devices in the extremely preterm population would be valuable.

Other limitations of this study included use of a term infant manikin. The simulation setting did not allow for testing device usability in the context of oral secretions, misting, infant movement, or glottic spasm. However, the simulation setting provided an environment to safely and systematically compare multiple devices using a comprehensive usability framework. The use of simulation also allowed us to examine of user experience with wider boundaries (e.g., for the time to intubation or number of attempts) than would be necessary if performing similar research safely and ethically in infants. Nevertheless, further evaluation of the usability of video laryngoscope devices in the clinical setting, including for resuscitation at birth and during subsequent neonatal intensive care, is warranted to guide selection video laryngoscopes for clinical use.

In this study, participants were highly experienced in neonatal intubation using conventional laryngoscopy, ensuring that their confidence and procedural skills were well established, reducing the risk that user experience with intubation would confound the comparison of usability. However, participants had varying experience using video laryngoscopes, and some of the devices were new to all participants. Further research with a focus on novices and those who infrequently intubate would give a broader perspective on device usability. Usability literature supports the need for repeated testing across diverse user groups to capture the full range of clinician experience [27].

## 5. Conclusions

Our study found that all six video laryngoscope devices were suitable for the intubation of a term neonatal manikin by experienced neonatologists, but their usability varied; the Koala Handheld, C-MAC, and GlideScope Miller devices demonstrated the highest usability, followed by the Koala Vision, GlideScope LoPro, and Parker. These findings highlight the importance of considering device usability when selecting video laryngoscopes for use. Further research involving novices and in the clinical setting is essential to further explore the usability of video laryngoscope devices and to better understand their impact on clinician performance and patient outcomes.

## Figures and Tables

**Table 1:** Participant demographics.

Participant Demographics	Frequency of Participants N (%)
Sex	2 (40%) Female 3 (60%) Male
Intubations with conventional laryngoscope	5 (100%) had completed > 100 conventional laryngoscope intubations
Intubations with video laryngoscope	1 (20%) no intubations with video laryngoscope 2 (40%) < 5 intubations with video laryngoscope 2 (40%) > 10 intubations with video laryngoscope
Previous experience with C-MAC	3 (60%) none 2 (40%) yes
Previous experience with GlideScope Miller	5 (100%) none
Previous experience with GlideScope LoPro	5 (100%) none
Previous experience with Koala Vision	2 (40%) none 3 (60%) yes
Previous experience with Koala Handheld	2 (40%) none 3 (60%) yes
Previous experience with Parker	2 (40%) none 3 (60%) yes
Self-rated competence to intubate with video laryngoscope	1 (20%) not competent 4 (80%) competent
Self-rated competence to supervise video laryngoscope intubation	3 (60%) not competent 2 (40%) competent

**Table 2:** Video laryngoscope devices characteristics.

**Video Laryngoscope**	**Blade**	**Screen and Mount**	**Image**	**Thematic Exemplar of Participant Comments (Participant Number)**
C-MAC^®^ (KARL STORZ, Brisbane, Australia)	Reusable Miller blades size 0, 1	8” screen unmounted	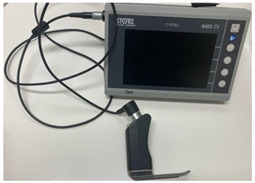	Ergonomics of Handle and Blade: “Thick handle doesn’t feel comfortable” (P4) “Size 1 is enormous. Blade is slim in the baby’s mouth” (P5) “Cord from screen to handle too long” (P2) Screen and Mount: “Screen can mount it on something. Would be better mounted” (P4) “Screen is very heavy, designed to be mounted. Would be worried without it mounted” (P2) Screen placed at the end of the bed near feet. Comparison to Conventional Laryngoscope: “Mental load of having to look down, would prefer just to look into the baby’s mouth to put the tube in” (P4)
GlideScope^®^ Core^TM^ system (GlideScope Miller) (Verathon, Brisbane, Australia)	Single-use Miller blades size 0, 1	10” screen mounted on stand with articulated arm	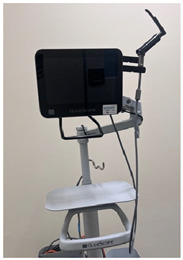	Ergonomics of Handle and Blade: “Weight of blade different” (P2) “Changing blade is easy” (P1) Screen and Mount: “Magnified picture” (P4) “Screen is fantastic, moves up and down” (P4) Stand is “bit bulky and heavy” (P1) Comparison to Conventional Laryngoscope: “Forgot to turn on prior to putting blade in mouth, because its open, looks like a regular laryngoscope jumped to that assumption” (P5)
GlideScope^®^ Core^TM^ system (GlideScope LoPro) (Verathon, Brisbane, Australia)	Single-use hyperangle LoPro blades size S1, S2	10” screen mounted on stand with articulated arm	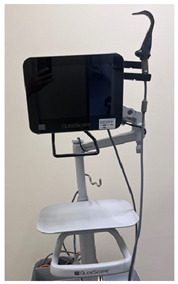	Ergonomics of Handle and Blade: “Never seen blade like this” (P4) “Just need to get angle right. Very angle dependent. If have right angle easy to do. Otherwise ETT goes into oesophagus” (P2) Screen and Mount: “Display is good. High quality. High resolution” (P3) “Screen allows movement up and down which is handy” (P4) Stand is “quite bulky” (P3) “Problem is positioning it during resuscitation” (P2) “Like video laryngoscope because magnifies view, makes easier for me” (P2)
Koala^®^ Vision Ultra (Koala Vision) (Koala Medical, Sydney, Australia)	Reusable Miller blades 00, 0, 1	8” screen mounted on stand	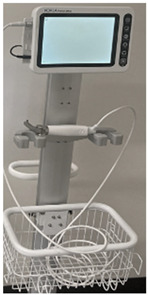	Ergonomics of Handle and Blade: “Quite good with 00 Miller” (P3) Screen and Mount: “Good resolution” (P3) “Can’t change the height…would like it lower” (P5) Stand is “quite easy to move” (P4) “Need to think about where to place with stand…Easier to move around than the GlideScope with stand” (P2) Comparison to Conventional Laryngoscope: “Similar to direct laryngoscope for changing blades” (P1)
Koala^®^ Handheld (Koala Medical, Sydney, Australia)	Reusable Miller blades 00, 0, 1	3.5” screen mounted on handle	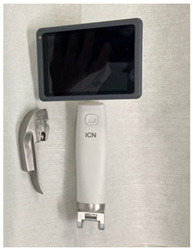	Ergonomics of Handle and Blade: “Feels like blade is really big in the mouth, big and wide” (P5) “Blade is similar to direct laryngoscope” (P1) Screen and Mount: “Like it because its compact” (P1) “I like that screen in field of vision” (P2) “Advantage not twisting to look at screen, everyone can see it.” (P3) “Top of handle can hit top of the cot” (P1) Teaching: Good for teaching because “instructor can see with magnification, can intubate directly” (P3) “Still use as teaching tool, get trainee close to look at screen” (P4) Comparison to Conventional Laryngoscope: “Similar to direct laryngoscope for changing blades” (P1) “Feels more intuitive because connected to blade and baby” (P5)
Parker Neonatal (Parker, Healthcare, Melbourne, Australia)	Reusable Miller blades sizes 000, 00, 0, 1	5” screen unmounted	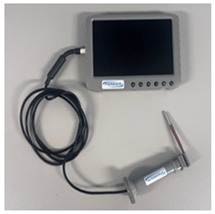 Parker Neonatal with size 1 blade without sleeve 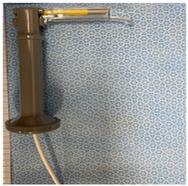 Size 00 blade with sleeve	Ergonomics of Handle and Blade: “Blade is small. Wouldn’t give good control of the tongue for difficult intubation” (P1) “They have really small blades 000, if 300-400g helpful as profile of blade is very small” (P2) “Handle not very ergonomical” (P4) “Change the blade matter of plugging in another blade… sleeve not intuitive… size once out of packaging not labelled, need to ensure correct sleeve attached” (P2) Screen and Mount: “Getting a lot of glare onto screen” (P5) “Screen fell over, would need assistant” (P3)

**Table 3:** System Usability Scale (SUS) for video laryngoscope devices.

Video Laryngoscope	SUS Median (IQR)
Parker	32.5 (23.8)
GlideScope LoPro	62.5 (23.8)
C-MAC	65.0 (10.0)
Koala Vision	72.5 (10.0)
GlideScope Miller	75.0 (22.5)
Koala Handheld	87.5 (25)

**Table 4:** NASA-TLX for video laryngoscope devices.

Video Laryngoscope	Overall NASA-TLX Median (IQR)	Mental Demand Median (IQR)	Physical Demand Median (IQR)	Temporal Demand Median (IQR)	Performance Median (IQR)	Effort Median (IQR)	Frustration Median (IQR)
Parker	50.8 (22.9)	60 (40)	60 (28)	30 (33)	25 (23)	60 (25)	55 (33)
GlideScope LoPro	45.0 (52.5)	65 (58)	50 (60)	35 (43)	40 (58)	40 (68)	20 (45)
Koala Vision	39.2 (33.8)	50 (58)	45 (35)	40 (50)	35 (23)	45 (48)	25 (33)
Koala Handheld	39.2 (30.4)	30 (48)	30 (38)	45 (35)	10 (33)	55 (55)	20 (33)
GlideScope Miller	32.5 (48.3)	35 (53)	50 (58)	30 (43)	20 (35)	35 (60)	20 (60)
C-MAC	26.7 (18.8)	20 (35)	35 (43)	30 (18)	15 (20)	25 (45)	20 (30)

**Table 5:** Efficacy and efficiency of video laryngoscope devices.

Video Laryngoscope	First-Pass Success N (%)	Number of Attempts Median (IQR)	Overall Success N (%)	Time to Successful Intubation Median (IQR)
GlideScope LoPro	4 (80%) *	1 (2.0) *	4 (80%) *	35.5 s (9.8) *
Koala Vision	4 (80%)	1 (1.0)	5 (100%)	31.0 s (14.0)
GlideScope Miller	4 (80%)	1 (1.0)	5 (100%)	25.0 s (10.5)
Parker	5 (100%)	1 (0.0)	5 (100%)	31.0 s (19.0)
Koala Handheld	5 (100%)	1 (0.0)	5 (100%)	26.0 s (12.5)
C-MAC	5 (100%)	1 (0.0)	5 (100%)	23.0 s (10.0)

* One participant was unable to perform intubation with GlideScope LoPro; their data are from think-aloud intubation attempts.

## Data Availability

The data presented in this study are available on request from the corresponding author due to privacy.

## Supplementary Materials

The following supporting information can be downloaded at https://www.mdpi.com/article/10.3390/children12060723/s1: S1: Detailed Study Procedure.

## Abbreviations

The following abbreviations are used in this manuscript:

SUS	System Usability Scale
NASA-TLX	National Aeronautics and Space Administration Task Load Index
IQR	Interquartile range
SPSS	Statistical Package for the Social Sciences
IPPV	Intermittent positive pressure ventilation
TT	Tracheal tube
ILCOR	International Liaison Committee on Resuscitation
P#	Participant number
