# Supplementary material for: Video Laryngoscopes in Simulated Neonatal Intubation: Usability Study
Source: Children (Basel). 2025 May 31;12(6):723. doi: 10.3390/children12060723 (PMC12191191; doi:10.3390/children12060723)
Supplement: Supplementary file 1 [file children-12-00723-s001.zip › children-3661613-supplementary.pdf]

Supplementary Material S1: Detailed Study Procedure

Step 1: Welcome and introduction to staff
Step 2: Information of study & Consent confirmed
Step 3: Orientation to simulation space and manikin
Step 4: Questionnaire: demographics
Step 5: Video laryngoscopes tested in randomised order- envelope opened

1st Video Laryngoscope:

Step 6: Orientation to video laryngoscope device:

- Size of blade that is attached
- How to turn on/off
- Operating on battery and does not need to be plugged in during use.
- Additional orientation for the GlideScope LoPro: "You may have experience with the low pro blade- it has a different shape to the Miller blades. You may be need to place the blade slightly to the left of the infant's mouth to allow room for the endotracheal tube (ETT) to pass, many find it easiest if the ETT is shapes as the blade is shaped, in a hockey stick formation".

Step 7: Experiments:

7.1 Observation

- This observation is about how you interact with the device
- Have you used this device before? Simulation or clinically?

7.2 Think Aloud Intubation

- Can you talk us through how you would use this device to intubate a newborn? Give us a running commentary. Whatever random thoughts pop in your head. It can be difficult to keep a commentary going, we may prompt you by saying 'keep going' throughout the simulation to encourage you to continue thinking aloud
- What was the experience of intubating with that device? What did you like? What didn't you like?

7.3 Function and Troubleshooting Tests:

- This simulation is for us to gain knowledge about the ease of function and troubleshooting the device.
- Device failure test: You go to use the video laryngoscope, the blade is in the mouth, the light is working, the screen goes black, would you proceed with the intubation?
- Would you use the video laryngoscope as a direct laryngoscope?
- You go to use the video laryngoscope, but it has a wrong size blade on. How do you change the blade?
- You want to record the footage of an intubation; how do you do this? Can you take a photo?
- You go to use this video laryngoscope, and the view is hazy what do you do?
- Do you check the view on the screen prior to inserting the blade into the mouth?

7.4 High-Fidelity Simulation:

- This simulation focuses on intubation using the video laryngoscope device.
- This simulation is for us to gain knowledge on the ease of use of the device.
- Vignette: "You are the neonatologist on, you are called to birth suite, a term baby has been delivered a few minutes ago and is apnoeic. On your arrival the neonatal nurse, is giving intermittent positive pressure ventilation via the mask to the baby. The baby remains apnoeic. You are the only two staff available for the resuscitation. The observations are available on screen. Please proceed."

Step 8: Complete the System Usability Scale (SUS) and National Aeronautics and Space Administration Task Load Index (NASA-TLX) questionnaires.

2nd- 6th Video Laryngoscope:

- Steps 6-8 completed for each video laryngoscope

Step 9: Semi-structured interview:

- Which Video Laryngoscope do you prefer? Why?
- Which type of laryngoscope would be your preference for a difficult airway – Traditional Laryngoscope or Video Laryngoscope? Which Video Laryngoscope?

- Which Video Laryngoscope do you think would be easiest for novice to learn on? Which Video Laryngoscope do you think would be easiest for you to provide supervision with?

Step 10: Debrief
